# Novel Insights into Prokineticin 1 Role in Pregnancy-related Diseases

**DOI:** 10.7150/ijms.76817

**Published:** 2024-01-01

**Authors:** Xi-Xi Cheng, Ming-Qing Li, Ting Peng

**Affiliations:** 1 Shanghai Changning Maternity & Infant Health Hospital, China.; 2 The Department of Obstetrics, Shanghai Obstetrics and Gynecology Hospital of Fudan University, Shanghai 200080, China.; 3 NHC Key Lab of Reproduction Regulation (Shanghai Institute of Planned Parenthood Research), Hospital of Obstetrics and Gynecology, Fudan University, Shanghai 200080, China.; 4 Shanghai Key Laboratory of Female Reproductive Endocrine Related Diseases, Hospital of Obstetrics and Gynecology, Fudan University, Shanghai 200080, China.; 5 Laboratory for Reproductive Immunology, Institute of Obstetrics and Gynecology, Hospital of Obstetrics and Gynecology, Fudan University, Shanghai 200080, China.

**Keywords:** prokineticin1, maternal-fetal interface, angiogenesis, recurrent miscarriage, preeclampsia, fetal growth restriction

## Abstract

Prokineticin 1 (PROK1) is a secreted protein involved in a range of physiological activities such as cell proliferation, migration, angiogenesis, and neuronal cell proliferation. Emerging evidences show that PROK1/PROK receptors (PROKRs) are expressed by trophoblasts, and decidual stroma cells at the maternal-fetal interface. PROK1 plays a critical role in successful pregnancy establishment by regulating the decidualization, implantation and placental development. Dysregulation of prokineticin signaling has been described in certain pathological states associated with pregnancy, including pre-eclampsia, recurrent miscarriage and fetal growth restriction. In this review, the expression and pleiotropic roles of PROK1 under physiological and pathological pregnancy conditions are discussed.

## 1. Introduction

Prokineticin family include two members, prokineticin 1 (PROK1; also called EG-VEGF) and prokineticin 2 (PROK2; also called Bv8). The *Human PROK1* gene maps to the regions of chromosome 1p13.1 and is composed of three exons with no alternative splicing product. The mature PROK1 protein is formulated of 86 amino acids and its relative molecular weight is 8.6 kDa 1. The *Human PROK2* gene maps to chromosome 3p21.1 2 and encodes a mature protein of 81 amino acid 1. PROKs are highly conserved in varies species, characterized by a conserved N-terminal sequence (AVITGA), which is essential for the correct binding of receptors 3.

These two secreted proteins act through two G protein-coupled receptors, prokineticin receptor 1 (PROKR1) and prokineticin receptor 2 (PROKR2) 4. PROKR1 is mainly involved in cell proliferation and angiogenesis, while PROKR2 is involved in the vital vascular endothelial permeability process. Receptors couple to Gq 5, Gi 6 and Gs 7 protein. Prokineticin receptors (PROKRs) mediate the intracellular calcium mobilization, phosphorylation of p44/p42 mitogen-activated protein kinase, serine/threonine kinase Akt and cyclic AMP (cAMP) accumulation, respectively 7. Additionally, PROKRs can either stimulate or inhibit the accumulation of cyclic AMP (cAMP) via Gs or Gi proteins 7, 8, respectively. Moreover, PROKRs have been shown to act through Gi protein to stimulate mitogen-activated protein kinase (MAPK) 6.

The PROK1 and PROK2 proteins are expressed in multiple organs and tissues, such as brain, heart, digestive tract, bone marrow, ovary, testis, decidua, and placenta. It was found that prokineticins showed dynamic expression throughout the physiological process in many organs, including circadian rhythm 9, menstrual cycle 10 and pregnancy 11. Additionally, prokineticins are involved in the cancer formation^12^.

The expression of prokineticin 1 in the reproductive system plays an important role in the development of the placenta. Prokineticin 1 could affect the growth and development of the placenta, including the processes of implantation 13, invasion and angiogenesis 11. Whereas the prokineticin 2 mainly affected in the central nervous system and could cause idiopathic hypogonadotropic hypogonadism by abnormal expression. The function of PROK2 in pregnancy-related disease is unclear and needed further study. In pregnancy-related diseases, PROK1 is related to preeclampsia 14, recurrent miscarriage 15, intrauterine growth restriction 14, premature birth 16 and other diseases. In this article, we are going to discuss the role of PROK1 in normal pregnancy and pregnant-related diseases.

## 2. Biological functions of the prokineticin1

Through constant study, researchers have identified many physiological functions of PROK1. The involvement of PROK1 in diverse physiological activities further demonstrates its important research value.

### 2.1 Prokineticin1 in ovary

Ovary is one of the organs where PROK1 protein highly expressed. Researchers discovered that *PROK1* was expressed in luteal steroidogenic cells of human ovaries. Luteal endothelial cells (LEC) expressed high levels of both PROKR1 and PROKR2 protein. PROK1 protein enhanced proliferation of luteal endothelial cells by increasing [3H]-thymidine binding, MAPK activation and c-jun/fos mRNA expression. The expression of PROKR2 protein is increased in LEC under some stress conditions,such as tumor necrosis factor α (TNFα) and chemical hypoxia,while the PROKR1 protein expression level is unchanged 17. Exogenous PROK1 protein can inhibit LEC apoptosis 17.

This implies that PROK1 may exert an anti-apoptotic effect on LEC through PROKR2. In addition, PROK1 increased *VEGF* mRNA expression in bovine luteinizing steroid-producing cells via PROKR1 17. These findings suggest that PROK1 plays an important role in luteal function by promoting LEC proliferation and anti-apoptotic effects 18. Researchers compared *PROK1* expression in 13 pairs of human polycystic ovary syndrome (PCOS) and normal ovary samples. *PROK1* was highly expressed in theca interna and stroma of PCOS ovaries, suggesting that the angiogenic function of PROK1 may be related to the cyst formation 19. In another study, researchers transfected rat ovarian granule cells with miR-28-5p mimics, and found that PROK1 protein level is downregulated 20. MiR-28-5p reverses the promotive effect of PROK1 on cell proliferation and the inhibitory effect on autophagy 20. PROK1 promoted proliferation and inhibited the apoptosis of rat ovarian granulosa cells through the AKT/mTOR signaling pathway 20.

Additionally, concentration of PROK1 protein in the follicular fluid is relevant to the follicular size and is predictive of the oocyte competence 21. Follicle Stimulating Hormone and hCG up-regulated PROK1 protein secretion in cumulus cell primary cultures, probably through the cAMP pathway 21. Successful embryo implantation may be predicted by PROK1 concentration in the follicular fluid and fertilization culture media in conventional in vitro fertilization-embryo transfer 22. Moreover, expression of *PROK1* and *PROK2* is correlative to the germ cell development in the human fetal ovary 23. PROK1 increases ERK phosphorylation and COX2 expression 23.

### 2.2 Prokineticin1 in gastrointestinal tract

PROK1 protein was originally identified as a promoter of gastrointestinal smooth muscle contraction 24. PROK1 has a high-affinity site in human ileal smooth muscle 24. Additionally,* PROKR1 mRNA* is highly expressed in rat ileum 25. In rat ileum, PROK1 induces a biphasic contractile response, including an early tetrodotoxin (TTX)-sensitive response and a late TTX-insensitive response 25.

*PROK1* mRNA is not expressed in the normal colorectal mucosa, but was detected in all colorectal cancer cell lines 26. Researchers found that PROK1 increased the microvascular density in colon cancer and promoted cancer cell proliferation 26. It also increased the liver metastatic ratio 26. A recent study showed that PROK1 was involved in lymphatic vessel production and participated in lymphatic metastatic pathways 27.

### 2.3 Prokineticin1 in the bone marrow and blood cells

In human or mouse hematopoietic stem cell cultures, PROK1 increases the number of granulocytic and monocytic colony-forming units 28. Systemic in vivo exposure to PROK1 significantly increased total leukocytes, neutrophils and monocytes 28. Moreover, PROK1 could protect multiple myeloma (MM) cells from apoptosis by upregulated Myeloid-cell-leukemia 1 (Mcl-1) 29. Furthermore, Mcl-1 was upregulated through MAPK, AKT and STAT3 pathway 29.

### 2.4 Prokineticin1 in neural crest cells

PROKR1 and PROKR2 are expressed in the human enteric neural crest cells (NCCs) 30, 31. PROK1 protein activates the Akt and MAPK pathways and induces proliferation and differentiation of NCCs via PROKR1 30. Neuroblastoma is derived from improperly differentiated neural crest cells. PROK1 induces neuroblastoma cell proliferation by activating the Akt pathway through PROKR1 and PROKR2 32. PROKR2 is essential for inhibiting neuroblastoma cell apoptosis 32.

## 3. The expression of PROK1 at the maternal-fetal interface

### 3.1 Endometrium

Immunohistochemistry and in situ hybridization analysis showed that PROK1, PROKR1 and PROKR2 were located in a variety of cellular components of the human uterus, including the glandular epithelium, stroma and endothelial cells of the endometrium, as well as in smooth muscle and endothelial cells of the myometrium 33 **(Table 1)**. Immunohistochemistry analysis showed that PROK1 was expressed primarily in the glandular epithelial cells and that its expression was dynamic during the menstrual cycle, and reached a peak in expression during the secretory phase^34^. *PROK1* mRNA expression in endometrial cells is controlled by E2 (estrogen) and P4 (progesterone) 34. Additionally, *PROK1* was rarely detected in the endometrial samples from the postmenopausal patients 34. This suggests that PROK1 expression is highly correlated with steroid hormones.

### 3.2 Decidua

The researchers found that PROK1 and its receptors PROKR1 and PROKR2 were expressed in decidual cells and play an important role in the process of endometrial decidualization 35. Expression of *PROK1* mRNA in human first-trimester decidua was significantly increased compared to non-pregnant endometrium 35.

### 3.3 Trophoblasts

The expression of *PROK1 mRNA* and *PROKR1 mRNA* peaks in trophoblast cells at 8-10 weeks of gestation 36. PROK1 protein is localized to the syncytiotrophoblasts 36, expressed minimally in cytotrophoblasts and not expressed in the extravillous trophoblasts 37. *PROKR2 mRNA* is not expressed in trophoblasts in the first trimester of pregnancy, except for low levels of expression detected at 8-10 weeks of gestation 37, 38. Moreover, PROKR1 protein is highly expressed in cytotrophoblasts, placental microvascular endothelial cells and Hofbauer cells, whereas PROKR2 protein is mainly expressed in syncytiotrophoblasts, Hofbauer cells and extravillous trophoblasts 37.

## 4. Regulation of prokineticin1 expression at maternal-fetal interface

### 4.1 Hormone

Researchers found that human chorionic gonadotropin (hCG) could induce the expression of PROK1 in a baboon model, human endometrial epithelial cells 39 and first-trimester decidua 39 (**Table 2**). They showed by dual immunohistochemical analysis that hCG receptors, PROK1 and PROKR1 were present in the glandular epithelial cells of the first trimester decidua 40. PROK1 and hCG show similar expression pattern during the first trimester of pregnancy. PROK1 expression peaks in trophoblast cells at 8-10 weeks of gestation, while the highest values of hCG are shown at 10-11 weeks of gestation^39^. Human chorionic gonadotropin increases the expression of PROK1, PROKR1 and PROKR2 mRNA and protein in dose- and time-dependent manners^39^. In another study, researchers found that primary endometrial stromal cells decidualization by progesterone and cyclic adenosine monophosphate could be inhibited by *PROK1* knockdown^39^. It is reported that hCG regulated PROK1 expression in the ovary 41. HCG can increase cAMP levels, and cAMP enhances PROK1 promoter expression^39^.

Battersby *et al*. 33 found that during menstrual cycle the expression of PROK1 protein in the endometrium showed periodic changes. The PROK1 protein showed higher expression in the secretory phase of the menstrual cycle than that in the proliferative phase 33. In endometrial cell culture, *PROK1* mRNA was detected in endometrial cells only in the presence of steroids 34. Using the porcine model of intrauterine infusions of estradiol-17 beta, researchers revealed that it upregulated endometrial expression of* PROK1* and* PROKR1* mRNA. Moreover, estradiol-17 beta, acting together with prostaglandin E2 (PGE2) increases PROKR1 protein expression in the endometrium 42.

Ujvary *et al*. 43 found that insulin upregulated PROK1 in a dose-dependent manner in decidualizing endometrial stromal cells. Furthermore, dihydrotestosterone is demonstrated to upregulate both of gene and protein expression of PROK1 along with insulin. But dihydrotestosterone alone do not show significant effects at all 44. On the one hand, insulin interacting with dihydrotestosterone enhances the decidualization, but on the other hand dysregulates the migration of decidual cells and the invasion of trophoblast cells 45.

### 4.2 Hypoxia

It was reported that hypoxia-inducible factor 1-alpha (HIF1α) response element was identified in the promoter region of PROK1 46. Researchers confirmed that *PROK1 mRNA* and *PROKR1 mRNA* were upregulated by hypoxia in the human placenta 37. Additionally, the stimulating effect of insulin on PROK1 gene and protein expression is mediated by HIF1α acting through the phosphatidylinositol 3-kinase (PI3K) signaling pathway 43.

### 4.3 Peroxisome proliferator-activated receptor-γ

Researchers confirmed that upregulating peroxisome proliferator-activated receptor-γ (PPARγ) increased the PROK1 and its receptors expression in both mRNA and protein level. On the other hand, the PPARγ^-/-^ mice showed low level expression of PROK1 47. It is shown that PPARγ plays an important role in the control of trophoblast migration and invasion 48. It was found that PPARγ upregulated PROK1 and inhibited trophoblast invasion via PROKR2 47.

## 5. Role of prokineticin1 in normal pregnancy

### 5.1 Regulation of trophoblast functions

The depth of invasion of placental trophoblast cells into the uterine wall is quite important in normal pregnancy. Therefore, during the placental development, the proliferation, differentiation and invasion of trophoblast cells are finely regulated. Diverse growth factors and cytokines stimulate differentiation of trophoblast cells to an invasive phenotype, such as epidermal growth factor (EGF), hepatocyte growth factor (HGF), transforming growth factor beta (TGF-β), insulin-like growth factor-II (IGF-II), and interleukin-1 (IL-1) 49.

**Figure 1:**
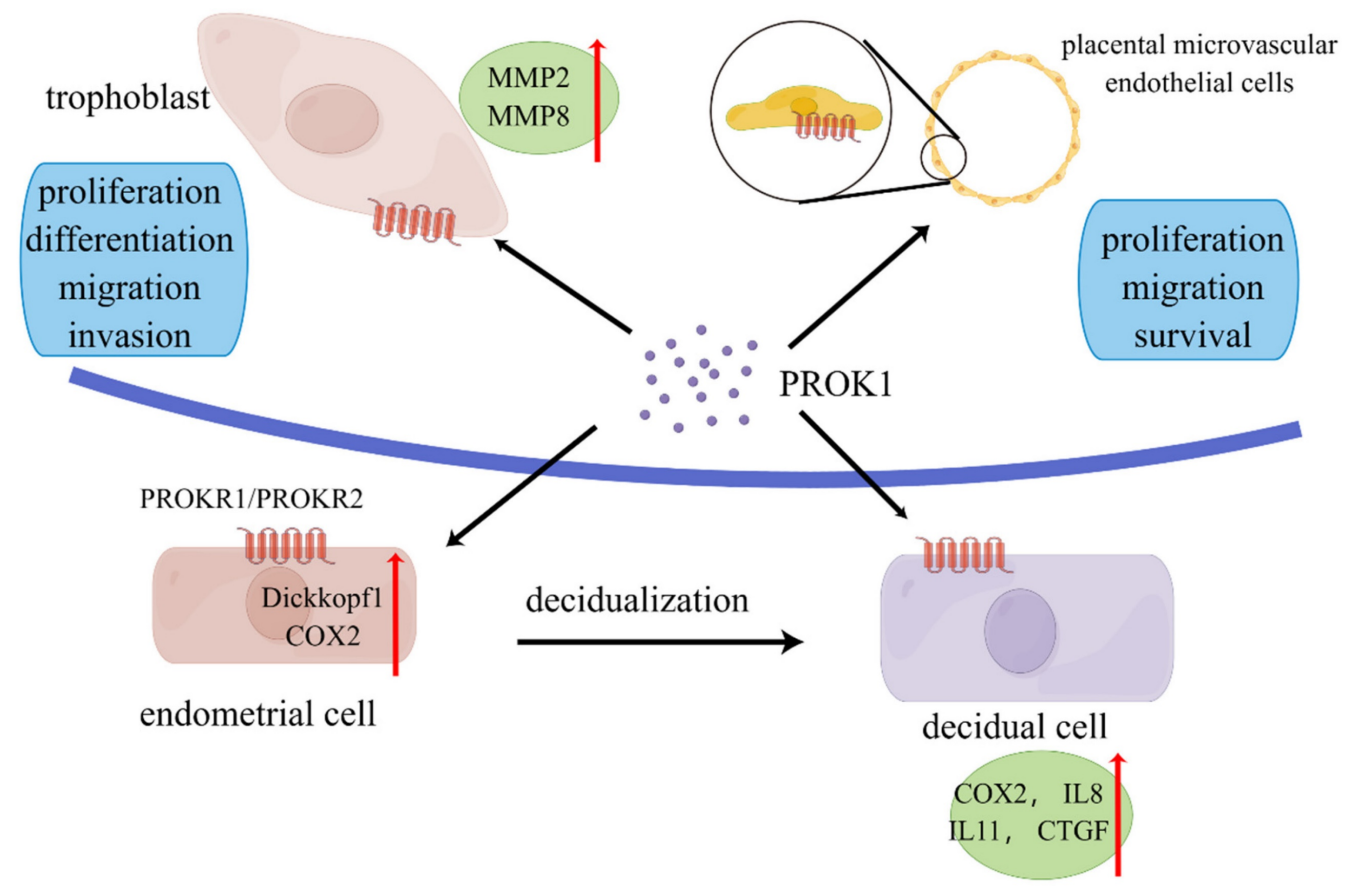
** Role of prokineticin1 in the maternal-fetal interface.** PROK is secreted mainly by placenta. PROK1 can promote proliferation, differentiation, migration and invasion of trophoblasts through upregulation of MMPs and other pathways. PROK1 acts on placental microvascular endothelium and affects angiogenesis. It acts on endothelial stromal cells and affects decidualization through upregulation of COX2, dickkopf1, etc. PROK1 affects placental implantation through regulation of IL8, IL11 and other cytokines. By Figdraw (www.figdraw.com).

Several studies have confirmed that PROK1 played an important role in trophoblast invasion, affecting trophoblast proliferation, migration, and invasion (**Figure 1**). Extravillous trophoblastic cells have an important role in the establishment of a successful pregnancy. These cells invade the decidua and spiral arteries to establish the fetal-mother circulation. PROK1 enhanced the invasion of extravillous trophoblastic cells (EVTs) by upregulating the expression of matrix metalloproteinases (MMP) 2 and MMP9 mRNA. And this upregulation was achieved through the receptor PROKR2 50. They also found the upregulation of extracellular signal-regulated kinase (ERK) 1/2 after the treatment of high concentration of PROK1 50. Researchers found that PROK1 activated ERK1/2 signaling and subsequent upregulation of MMP2 and MMP9 mRNA levels, thereby stimulating invasion of human trophoblast HTR-8/Svneo cells 51. Therefore, the decrease in PROK1 circulating and placental levels at the end of the first trimester may, with other factors, contribute to EVT invasion and to the establishment of fetal-maternal circulation 52.

Primary cilia are cellular antennae that receive environmental signals and are essential for normal development. PROK1 and its receptors were detected in primary cilia 51. It was found that inhibition of primary cilia growth could cause decrease of PROK1 and MMP9 expression 51. In a study published recently, researchers showed that miR-346 and miR-582-3p regulated PROK1-induced trophoblast invasion through repressing MMP 2 and MMP 9^53^. It was shown that miR-346 and miR-582-3p inhibited not only PROK1 expression but also trophoblast invasion and migration in JAR and HTR-8/Svneo cell lines^53^. The PROK1-stimulated cell proliferation was mediated by PI3K/AKT/mTOR, MAPK, and cAMP 13. PROK1 enhanced adhesion of trophoblast cells to fibronectin and laminin matrices, which are mediated predominantly via leukemia inhibitory factor (LIF) induction 40.

### 5.2 Effect on endometrium/decidua

Decidualization is a complex process. Endometrial stromal cells are regulated by hormones as well as cytokines and undergo morphological, functional and genetic changes to support embryo implantation and development 54. PROK1 and its receptors regulate the implantation and decidualization via affecting the expression of implantation-related genes. It has been shown that PROK1 can regulate the expression of many genes, including: LIF, COX-2, IL-6, IL8, and IL-11 35, 42. These cytokines are verified to be related to the implantation and decidualization.

Differentiation of endometrial stromal cells into decidua is mediated by PGE2 synthesis through elevation of COX-2 protein 35, 55. In an experimentation conducted by Sharon *et al*, the expression of COX-2 protein was significantly increased after the treatment of PROK1 in a human germ cell tumor line. And it was confirmed that PROK1 signaling via PROKR1 induce the expression of COX-2 protein^23^. Another research showed that PROK1 induced COX-2 expression and prostaglandin synthesis in human endometrial cells and first trimester decidua via a Gq coupled pathway 56. Another study found that PROK1 could upregulate the Dickkocf1 which regulate cells proliferation and decidualization of endometrial stromal cells. It was confirmed through a Gq-calcium-calcineurin-nuclear factor of activated T-cells-mediated pathway 57.

IL-8 is highly expressed in the endometrium and decidua and involves in several processes of endometrial physiology such as angiogenesis, proliferation, chemotaxis, trophoblast invasion and uterine contraction. Researchers found that in endometrial cells PROK1 via PROKR1 could activate the calcineurin/NFAT pathway to induce IL-8 expression 58.

In human endometrial stromal cells, IL-11 has been shown to promote progesterone-induced decidualization, which implies a role for IL-11 in preparing the endometrium for implantation 59. PROK1-PROKR1 induces the expression of IL-11 in Ishikawa cells and first trimester decidua in a guanine nucleotide-binding protein (G(q/11)), extracellular signal-regulated kinases, Ca^2+^ and calcineurin-nuclear factor of activated T cells dependent manner through the calcium-calcineurin signaling pathway 60.

Connective tissue growth factor is considered to be up-regulated by PROK1 in the first-trimester decidua^61^. PROK1 regulates the expression of connective tissue growth factor by activating the Gq, PLC, cSrc, EGFR, MAPK/ERK kinase pathway^61^.

### 5.3 Effect on angiogenesis

Several studies have elucidated the angiogenic role of PROK1 in reproductive organs. Study found that PROK1 mainly acted on human placental microvascular endothelial cells (HPEC), and stimulated HPEC's proliferation, migration and survival, while VEGF mainly affected the umbilical vein-derived macrovascular endothelial cells 62. Compared to VEGF, PROK1 was more effective in stimulating HPEC sprout formation, pseudovascular organization, and it significantly promoted HPEC permeability and paracellular transport 62. In another study, researchers demonstrated that PROK1 controlled homeobox genes expression in normal human placenta and in placenta from fetal growth restriction (FGR) pregnancies 63. The homeobox genes expressed in placental microvascular endothelial cells may play a role in the regulation of epithelial-mesenchymal interactions in the placenta and proliferative capacity of placental microvascular endothelial cells 64.

The effects of PROK1 on HPECs suggests that this factor is involved in placental angiogenesis, a process closely related to placental growth and fetal maternal exchanges.

## 6. Role of prokineticin1 in pathological pregnancy

The dynamic expression of PROK1 throughout pregnancy, its multiple roles in the villi development, and its regulation by hypoxia suggest that this cytokine contributes to the successful pregnancy establishment. Thus, inappropriate expression and/or dysfunction of PROK1 may lead to major complications of pregnancy (**Table 3**), such as recurrent implantation failure, recurrent pregnancy loss (RPL), ectopic pregnancy (EP), fetal growth restriction (FGR), choriocarcinoma, and pre-eclampsia (PE). These complications are associated with abnormal angiogenesis, as well as trophoblast invasion failures and abnormal placental development.

### 6.1 Pre-eclampsia

Pre-eclampsia is the most common hypertensive disease during pregnancy. There are several theories about the pathogenesis of pre-eclampsia. Although the etiology of PE is far from being fully understood, its development is thought to be primarily due to superficial invasion of the maternal decidua and spiral arteries by EVTs 65. It creates a prolonged hypoxic environment in the placenta, and leads to the release of antiangiogenic factors from the placenta 65. More specific and earlier markers associated with the disease need to be further investigated. PROK1 satisfies many of the characteristics of an early PE markers. It is abundant in the human placenta during early pregnancy, its expression is elevated by hypoxia, it controls trophoblast invasion, and has specific effects on placental endothelial cells. More importantly, PROK1 inhibit the EVTs which may play a critical role in the cause of PE.

Scientists found that the RNA and protein expression levels of PROK1 were significantly elevated in PE patients compared with age-matched control 10. The RNA and protein expression of PROK1 in the placenta of PE patients decreases significantly, suggesting that the in-situ expression of PROK1 in the placenta of PE patients may be down regulated at the transcriptional level 66. Moreover, the RNA and protein expression level of PROKR1 shows no difference between control and PE patients 66.

A group created a new mouse model of gestational hypertension by continuous injection of prokineticin1 14. Sustained high levels of ghrelin over the first trimester of pregnancy significantly increased blood pressure and renal impairment in mice 14. It significantly decreased the thickness of the decidual and junctional zone in the placenta 14. Moreover, sustained high levels of prokineticin1 in late pregnancy reduced blood pressure in mice compared to mice injected only up to mid-gestation 14.

Both of the RNA and protein expression of PROK1 is upregulated by hypoxia. The promoter regions of the gene contain functional hypoxia response elements that bind to hypoxia-inducible factor 1 (HIF-1) and mediate its expression under hypoxic tension. PROK1 upregulation by hypoxia confirms its role in the first trimester, as placental development and angiogenesis occur in hypoxic environment during this period 67.

The miRNA-200 family is predicted to target the PROK1 5'-untranslated region 68. Increased miRNA-141 and miRNA-200a inhibit the protein expression of PROK1, matrix metalloproteinase 9 (MMP9) and downstream extracellular signal-regulated kinase (ERK) signaling, thus leading to trophoblast invasion failures 68. The growth of the primary cilium is inhibited by increases in miR-141 and miR-200a expression 68. The number of cilia in the human placenta of preeclampsia women was markedly decreased compared to normal placenta 68.

### 6.2 Recurrent miscarriage

Recurrent miscarriage is related to abnormal blood vessels in the placental bed, suggesting that early disturbance of placental blood vessel development may lead to miscarriage 69.

In 2010, Su *et al*. 15 studied the genes of 115 women with recurrent miscarriage and 170 women from the control group, and selected 11 single nucleotide polymorphisms from PROK1, PROKR1, and PROKR2 by using genotype analysis. Further using multifactor dimensionality reduction (MDR) method for analysis, they found that 2 SNPs of PROKR1 (rs4627609, rs6731838) and 1 SNP of PROKR2 (rs6053283) are closely related to recurrent miscarriage (P<0.05) 15. At the same time, the frequency of the C-G and T-A haplotypes of PROKR1 and the A-G-C-G-G haplotypes of PROKR2 were also significantly increased in recurrent miscarriage (P <0.05) 15.

Hence, the gene polymorphism and haplotype of PROK1 receptor are both related to recurrent miscarriage. In a study carried out in 2014, researchers found the PROKR1 (I379V) and PROKR2 (V331M) were nonsynonymous variants showed significant association with recurrent miscarriage 70. It has been shown that the common variant of V67I may act as a modifier in the PROK1-PROKR system through down-regulation of PROK1 expression 71. PROK1 (V67I) has been shown to play a role as a modifier gene in the PROK1-PROKR system of human early pregnancy 71.

### 6.3 Recurrent implantation failure

In a recent study, researchers studied the endometrial samples of 15 women with recurrent implantation failure (RIF) and 15 women from control group. The results showed that *PROK1 mRNA* levels were 6.09 times higher compared to endometrial samples obtained from women with RIF than in samples obtained from women from control group, whereas *PROKR1 mRNA* levels were 2.46 times lower in endometrial samples obtained from women with RIF than in samples from women from control group. There was no statistically significant difference between women with RIF and women from control group regarding *PROK2* and *PROKR2* levels 72. The results suggested that expression of the PROK1/PROKR1 system could play an important role in implantation.

### 6.4 Fetal growth restriction

During pregnancy, fetal optimal growth depends on an adequate blood vascular network in the fetal part. Abnormal placental angiogenesis will endanger the supply of nutrients and hormones, which will eventually lead to FGR. Although the exact cause of FGR in not clear yet, several researches shows that the PROK1 and its receptors are highly associated with FGR. Scientists found that in FGR patients in the third trimester of pregnancy, both RNA and protein levels of PROK1 are significantly increased in the placenta 73. Scientists found that PROK1, PROKR1, and PROKR2 mRNA and protein levels were significantly increased in FGR placentas 73. PROK1 can increase the angiogenesis of placental villi and the expression of CD31, a marker of endothelial cells in the placental villi. At the same time, PROK1 promotes the proliferation of villi and the anchoring of cytotrophoblast layers, and promotes the survival of placental villi under stress conditions 73. Therefore, the increase of PROK1, PROKR1 and PROKR2 occurs in FGR as a compensatory mechanism to ensure proper pregnancy progression.

### 6.5 Choriocarcinoma

It has been suggested that the development and progression of choriocarcinoma is associated with an over-proliferation of the trophoblastic layer, resulting in an increased mass of cells that acquire a migratory and/or aggressive phenotype 74. Prokineticin 1 is demonstrated to upregulate trophoblast proliferation and invasion 73. Prokineticin 1 protein and its two receptors are upregulated in the blood and placenta of choriocarcinoma patients 75. Additionally, in the mouse model of choriocarcinoma, the application of PROKR1 or PROKR2 antagonists to gravid mice delayed tumor progression and increased the duration of pregnancy maintenance 75.

## 7. Conclusions

During the past 20 years, the prokineticin system has been reported to play a role in a range of physiological functions. Mutations or dysfunction of these factors are also associated with a number of diseases. Targeting prokineticin system may provide treatments for diseases such as cancer, inflammation, pain, and neuropathology. In recent years, more and more concerns have been expressed about their specific role at the maternal-fetal interface. Since the PROK2 is mainly expressed in the central nervous system and PROK1 is widely detected in the steroidogenic organs, researches mainly focused on the PROK1 and further research is needed to reveal the functions of PROK2 in the maternal-fetal interface.

PROK1 is co-regulated by pregnancy-associated hormones, cytokines and other factors. It is highly expressed in embryonic trophoblasts and decidual stromal cells at the maternal-fetal interface, and participates in a series of physiological processes through interaction with its receptors, PROKR1 and PROKR2. PROK1 not only promotes the endometrial decidualization process and enhances uterine receptivity, but also induces migration and invasion of trophoblasts and facilitate embryo implantation and development. Nevertheless, abnormal expression of PROK1 may lead to impaired endometrial decidualization, trophoblastic dysfunction and placental dysplasia, resulting in pregnancy complications such as recurrent miscarriage, PE and FGR, although the exact mechanisms have not been fully clarified. Therefore, normal expression of PROK1 in the first trimester is essential for a successful pregnancy. Regulation of PROK1, PROKR1 and PROKR2 expression may be potential targets for clinical therapy of patients with abnormal pregnancies, although further studies are needed to confirm this.

## Figures and Tables

**Table 1:** The expression of PROK1, PROKR1, and PROKR2 at maternal-fetal interface

Site	Cell	PROK1	PROKR1	PROKR2	Reference
Endometrium	Glandular epithelial cell	+*	+	Weak	26
	Stromal cell	+	+	-	26
	Endothelial cell in the endometrium or Myometrium	+	+	Weak	26
	Smooth muscle cell	+	+	-	26
Decidua	Decidual cell	+	+	+	35
Placenta	Syncytiotrophoblast	+	+	+	10,37
	Cytotrophoblast	+	+	Weak	10,36
	Extravillous trophoblast		Weak	+	10
	Placental microvascular endothelial cells	+	+		36
	Placental endothelial cells		-	+	10
	Macrophages		+	+	10,36

*: PROK1 is expressed primarily in the glandular epithelial cells.

**Table 2:** Regulation the expression of PROK1, PROKR1, and PROKR2 at maternal-fetal interface

Hormone	PROK1	PROKR1	PROKR2	Reference
Human chorionic gonadotropin	+	+	+	39
Progesterone	+	+		11,26,34
Estrogen	+	+		42
Dihydrotestosterone*	+			44
Insulin	+			43
Hypoxia	+	+		37
PPARγ	+	+	+	47

* Only effect with insulin.

**Table 3:** The role of PROK1 in pregnant related disease

Disease	Abnormal expression of PROK1	Functions of PROK1	Reference
Pre-eclampsia	higher	Inhibit trophoblast invasion	10,66, 67
Recurrent miscarriage	lower	gene polymorphisms of PROK1 and PROKR1 are related to recurrent miscarriage	15,70,71
Recurrent implantation failure	higher	unclear	72
Fetal growth restriction	high	Increase the angiogenesis and proliferation of placental villi	73

## Abbreviations

PROK1: prokineticin1
PROKR: prokineticin receptor
EG-VEGF: endocrine gland-derived vascular endothelial growth factor
cAMP: cyclic AMP
MAPK: mitogen-activated protein kinase
AKT: proto-oncogene protein c-akt
LEC: luteal endothelial cells
TNFα: tumor necrosis factor α
PCOS: polycystic ovary syndrome
Mcl-1: Myeloid-cell-leukemia 1
MM: multiple myeloma
hCG: human chorionic gonadotropin
PGE2: prostaglandin E2
HIF1α: hypoxia-inducible factor 1-alpha
PI3K: phosphatidylinositol 3-kinase
PPARγ: peroxisome proliferator-activated receptor-γ
EGF: epidermal growth factor
HGF: hepatocyte growth factor
TGF-β: transforming growth factor beta
IGF-II: insulin-like growth factor-II
IL-1: interleukin-1
EVTs: extravillous trophoblastic cells
MMP: matrix metalloproteinases
ERK: extracellular signal-regulated kinase
LIF: leukemia inhibitory factor
G(q/11): guanine nucleotide-binding protein
HPEC: human placental microvascular endothelial cells
RPL: recurrent pregnancy loss
EP: ectopic pregnancy
FGR: fetal growth restriction
PE: pre-eclampsia
RIF: recurrent implantation failure
